# Disarming *Pseudomonas aeruginosa* Virulence by the Inhibitory Action of 1,10-Phenanthroline-5,6-Dione-Based Compounds: Elastase B (LasB) as a Chemotherapeutic Target

**DOI:** 10.3389/fmicb.2019.01701

**Published:** 2019-08-02

**Authors:** Anna Clara M. Galdino, Lívia Viganor, Alexandre A. de Castro, Elaine F. F. da Cunha, Thaís P. Mello, Larissa M. Mattos, Marcos D. Pereira, Mary C. Hunt, Megan O’Shaughnessy, Orla Howe, Michael Devereux, Malachy McCann, Teodorico C. Ramalho, Marta H. Branquinha, André L. S. Santos

**Affiliations:** ^1^Department of General Microbiology, Institute of Microbiology Paulo de Góes, Federal University of Rio de Janeiro, Rio de Janeiro, Brazil; ^2^Postgraduate Program in Biochemistry, Institute of Chemistry, Federal University of Rio de Janeiro, Rio de Janeiro, Brazil; ^3^The Centre for Biomimetic and Therapeutic Research, Focas Research Institute, Technological University Dublin, Dublin, Ireland; ^4^Department of Chemistry, Federal University of Lavras, Lavras, Brazil; ^5^Department of Chemistry, Maynooth University, Maynooth, Ireland

**Keywords:** *Pseudomonas aeruginosa*, elastase B, metal-based compounds, 1,10-phenanthroline-5,6-dione, anti-virulence therapy, theoretical calculations

## Abstract

Elastase B (lasB) is a multifunctional metalloenzyme secreted by the gram-negative pathogen *Pseudomonas aeruginosa*, and this enzyme orchestrates several physiopathological events during bacteria-host interplays. LasB is considered to be a potential target for the development of an innovative chemotherapeutic approach, especially against multidrug-resistant strains. Recently, our group showed that 1,10-phenanthroline-5,6-dione (phendione), [Ag(phendione)_2_]ClO_4_ (Ag-phendione) and [Cu(phendione)_3_](ClO_4_)_2_.4H_2_O (Cu-phendione) had anti-*P. aeruginosa* action against both planktonic- and biofilm-growing cells. In the present work, we have evaluated the effects of these compounds on the (i) interaction with the lasB active site using *in silico* approaches, (ii) lasB proteolytic activity by using a specific fluorogenic peptide substrate, (iii) *lasB* gene expression by real time-polymerase chain reaction, (iv) lasB protein secretion by immunoblotting, (v) ability to block the damages induced by lasB on a monolayer of lung epithelial cells, and (vi) survivability of *Galleria mellonella* larvae after being challenged with purified lasB and lasB-rich bacterial secretions. Molecular docking analyses revealed that phendione and its Ag^+^ and Cu^2+^ complexes were able to interact with the amino acids forming the active site of lasB, particularly Cu-phendione which exhibited the most favorable interaction energy parameters. Additionally, the test compounds were effective inhibitors of lasB activity, blocking the *in vitro* cleavage of the peptide substrate, aminobenzyl-Ala-Gly-Leu-Ala-*p*-nitrobenzylamide, with Cu-phendione having the best inhibitory action (K*_*i*_* = 90 nM). Treating living bacteria with a sub-inhibitory concentration (½ × MIC value) of the test compounds caused a significant reduction in the expression of the *lasB* gene as well as its mature protein production/secretion. Further, Ag-phendione and Cu-phendione offered protective action for lung epithelial cells, reducing the A549 monolayer damage by approximately 32 and 42%, respectively. Interestingly, Cu-phendione mitigated the toxic effect of both purified lasB molecules and lasB-containing bacterial secretions in the *in vivo* model, increasing the survival time of *G. mellonella* larvae. Collectively, these data reinforce the concept of lasB being a veritable therapeutic target and phendione-based compounds (mainly Cu-phendione) being prospective anti-virulence drugs against *P. aeruginosa*.

## Introduction

The effectiveness of antimicrobial therapy to treat bacterial infectious diseases has been compromised by the emergence of multidrug-resistant (MDR) strains (Brown and Wright, 2016). The failure of classical antibiotic approaches represents a growing threat to public health worldwide. It has been estimated that approximately 50,000 deaths per year within the United States and Europe are due to antibiotic-resistant microbial infections (Ali et al., 2018). Additionally, the infections caused by antibiotic, non-responsive pathogens have prompted the substantial rise in healthcare expenditures (Ventola, 2015). According to the World Health Organization (Tacconelli et al., 2018), the cost for the treatment of MDR infections in the European Union exceeds 1.5 billion annually. In this respect, the treatment of *Pseudomonas aeruginosa* infections is particularly challenging due to the increasing global prevalence of MDR strains (Sonmezer et al., 2016). This gram-negative bacterium is armed with a sophisticated genetic machinery, presenting a plasticity arsenal of antimicrobial resistance mechanisms (Murray et al., 2015; Ali et al., 2018). Also, *P. aeruginosa* can quickly develop resistance to antimicrobial drugs through the acquisition of resistance genes in mobile genetic elements (Ali et al., 2018). According to the surveillance study conducted by the International Nosocomial Infection Control Consortium (INICC), which analyzes the antimicrobial susceptibility profile of strains recovered from patients admitted at intensive care units in Latin America, Europe, Eastern Mediterranean, Southeast Asia and Western Pacific countries, 29.9 and 44.3% of blood-cultured *P. aeruginosa* strains were resistant to amikacin and imipenem, respectively (Rosenthal et al., 2016).

*Pseudomonas aeruginosa* has a large range of extracellular virulence attributes (e.g., proteases, pyocyanin, exotoxins, and lipases), which are responsible for increasing its pathogenicity (Balasubramanian et al., 2013). Collectively, the pseudomonal virulence attributes enable the bacterium to (i) overcome host immune defenses, (ii) cause host damages, and (iii) induce dysfunctional alterations on the bacterial physiology that favors the establishment and maintenance of an infectious process (Azam and Khan, 2018). In this context, proteases are the protagonists of numerous physiopathological events in *P. aeruginosa*. Corroborating this statement, 3% of the open read frames in the whole *P. aeruginosa* genome are responsible for encoding proteases, which gives the bacterium the ability to adapt its sophisticated enzymatic machinery according to both the environment and to challenging conditions (Stover et al., 2000).

Multifunctional elastase B (lasB or pseudolysin) is the most abundant protease harvested from pseudomonal secretome, and it has been extensively studied (Galdino et al., 2017). LasB is a neutral, metallo-type (Zn^2+^/Ca^2+^ cofactors) protease, whose synthesis is guided by the expression of the *lasB* gene and is regulated by the *quorum sensing* (QS) transcription systems, *las* and *rhl* (Han et al., 2014). LasB is the protagonist molecule in the acute stage of *P. aeruginosa* infections as it degrades the extracellular matrix constituents (e.g., elastin, collagen types III and IV, laminin, fibronectin, and vitronectin) of host cells, inducing tissue injury and hemorrhage (Yang et al., 2015). Also, lasB displays relevant actions during chronic infections through its ability to manipulate host responses (Suarez-Cuartin et al., 2017) by degrading several components of the immune defenses (e.g., tumor necrosis factor-α, interferon-γ and interleukin-2) and by inactivating cell-bound C1 and C3 and fluid-phase C5, C8 and C9 components of the complement system (Kida et al., 2008; Kuang et al., 2011). Corroborating the relevance of lasB in the pathogenesis of *P. aeruginosa*, it was shown that rabbits infected with a Δ*lasB* mutant strain exhibited reduced severity of corneal ulceration (Cowell et al., 2003). Likewise, the deletion of the *lasB* gene resulted in a less invasive *in vivo* infection in both mouse and *Caenorhabditis elegans* models when compared to the infection caused by the respective wild-type strain (Tang et al., 1996; Tan et al., 1999).

To overcome the rising threat of MDR *P. aeruginosa*, the development of innovative chemotherapy is of immediate necessity (Tacconelli et al., 2018). Consequently, some chemists have focused their attention on the design and synthesis of potential metal-based drug molecules (McCann et al., 2012a). Metal-based antibiotics hold great promise due to their reactivity with bacterial cells, presenting multi-target and versatile mechanisms of action (Lemire et al., 2013). Our interdisciplinary research team has synthetized novel antimicrobial drug candidates based on 1,10-phenanthroline-5,6-dione (phendione) coordinated to transition metals (McCann et al., 2004). For instance, Ag^+^- and Cu^2+^-phendione complexes presented potent anti-*P. aeruginosa* action, inhibiting both planktonic- and biofilm-growing cells (Viganor et al., 2016).

Faced with the upcoming post-antibiotic era, the anti-virulence approach emerges as a promising strategy against superbug infections as it acts by disarming the virulence arsenal of the pathogen and, consequently, reducing the ability of the organism to damage host cells (Allen et al., 2014; Dickey et al., 2017). Therefore, this therapeutic strategy reduces the selective pressure on bacterial cells as well as delaying the emergence of non-responsive strains, since it does not affect the bacterial proliferation mechanisms (Allen et al., 2014). Hence, in view of the pivotal role of lasB in *P. aeruginosa* pathogenesis, the inactivation of this multifunctional enzyme is seemingly a promising target in anti-*P. aeruginosa* drug design. In this sense, phendione is chemically derived from 1,10-phenanthroline, which is a potent inhibitor of metallo-type proteases, including *P. aeruginosa* lasB (Galdino et al., 2017). Herein, we have evaluated the effects of phendione and its Cu^2+^ and Ag^+^ complexes on the (i) interaction with the amino acids forming the lasB active site, (ii) lasB proteolytic activity, (iii) *lasB* gene expression, (iv) lasB protein secretion, (v) ability to block the damages induced by lasB on a lung epithelial cell line, and (vi) survival of *Galleria mellonella* larvae challenged with purified lasB molecules and lasB-containing bacterial secretions.

## Materials and Methods

### Compounds

**FIGURE 1:**
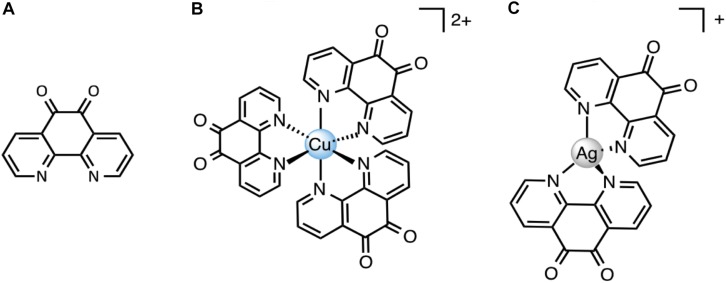
Chemical structures of 1,10-phenantroline-5,6-dione (phendione) **(A)**, [Cu(phendione)_3_](ClO_4_)_2_.4H_2_O (Cu-phendione) **(B)** and [Ag(phendione)_2_]ClO_4_ (Ag-phendione) **(C)**. Perchlorate anions and water molecules have been omitted for clarity.

1,10-Phenanthroline-5,6-dione (phendione), [Ag(phendione)_2_] ClO_4_ (Ag-phendione) and [Cu(phendione)_3_](ClO_4_)_2_.4H_2_O (Cu-phendione) (Figure 1) were synthetized in accordance to the methods previously described in the literature (McCann et al., 2004). The compounds were dissolved in dimethylsulfoxide (DMSO) at 100 mM, then the solutions were sterilized by filtration through a 0.22 μm membrane and stored at room temperature in the dark.

### *In silico* Analyses

#### Docking Procedure

In this theoretical analysis, docking calculations were employed in order to (i) identify favorable structural features of phendione, Ag-phendione and Cu-phendione through calculation of interaction energies with the known *P. aeruginosa* lasB inhibitor ligand, *N*-(1-carboxy-3-phenylpropyl)-phenylalanyl-α-asparagine (HPI) [employing Molegro Virtual Docker software (MVD^®^)], and (ii) calculate their hydrogen bonding interaction energies with amino acid residues within the cavity of the lasB enzyme (employing Semi-empiric-PM6 calculation).

The chemical structure of each phendione-based test compound was constructed and optimized at the semi-empirical level by employing the PC Spartan Pro program (Hehre et al., 1999). The partial charges on the atoms were also elucidated. The phendione-based test compounds were then docked inside the *P. aeruginosa* lasB crystallographic structure containing the HPI inhibitor (PDB code: 1U4G; resolution: 1.4 Å) (Thayer et al., 1991) by using the Molegro Virtual Docker software (MVD^®^) (Thomsen and Christensen, 2006), taking into account the same procedures employed in previous works (Silva et al., 2015; Soares et al., 2018). For the development of the docking calculations, a radius of 5 Å centered at the active cavity was considered, where the residues were kept flexible. Due to the nature of the employed docking methods, the calculations were carried out generating 50 poses (conformation and orientation) for each phendione-based test compound.

The MolDock scoring function employed in the MVD program comes from the piecewise linear potential (PLP), a simplified potential whose parameters are fitted to protein-ligand structures, binding data scoring functions and further extended in the Generic Evolutionary Method for molecular docking with a new hydrogen binding term and new charge schemes (Goncalves et al., 2014). Next, Equation (1) defines the docking scoring function values (Escore):

(1) $${\text{E}}_{\text{score}}={\text{E}}_{\text{inter}}+{\text{E}}_{\text{intra}}$$

Wherein:

(2) $$E_{\text{inter}}=\sum_{i\,s\,l\,i\,g\,a\,n\,d}\sum_{j\,s\,p\,r\,o\,t\,e\,i\,n}\left[E_{P\,L\,P}\,\left(r_{i\,j}\right)+332.0\,\frac{q\,i\,q\,j}{4\,r_{i\,j}^{2}}\right]$$

E_*PLP*_ (piecewise linear potential) consists of two different parameter sets: one for the approximation of the steric term (van der Waals) among the atoms, and the other potential for the hydrogen binding. The second term is related to the electrostatic interactions among overloaded atoms. It is a Coulomb potential with a dielectric constant dependent on the distance (D(r) = 4r). The numerical value of 332.0 is assigned for the electrostatic energy (in kcal mol^–1^) (Thomsen and Christensen, 2006). E_*intra*_ is the internal energy of the ligand (Equation 3):

(3) $$E_{i\,n\,t\,\text{ra}}=\sum_{i\,s\,l\,i\,g\,a\,n\,d}\sum_{j\,s\,l\,i\,g\,a\,n\,d}E_{P\,L\,P}\,\left(r_{i\,j}\right)+\sum_{f\,l\,e\,x\,i\,b\,o\,n\,d\,s}A\left[1-\cos\left(m.\theta -\theta_{0}\right)\right]+E_{c\,l\,a\,s\,h}$$

The first part of the equation (double summation) is among all pairs of atoms in the ligand, taking off those that are connected by two bonds. The second one characterizes the torsional energy, wherein *θ* is the torsional angle of the bond. If several torsions could be determined, each torsional energy is considered and the average value used. The last term, E_*clash*_, assigns a penalty of 1,000 if the distance between two heavy atoms (more than two bonds apart) is smaller than 2.0 Å, disregarding infeasible ligand conformations (Thomsen and Christensen, 2006). The docking search algorithm that is applied in the MVD program algorithm considers interactive optimization techniques that are inspired by considering an evolutionary Darwinian theory and a new hybrid search algorithm called guided differential evolution. This hybrid combines the differential evolution optimization technique with a cavity prediction algorithm during the search process, thus permitting a fast and accurate identification of potential binding modes (poses) (Thomsen and Christensen, 2006).

#### Semi-Empirical Methods

In this stage, a further theoretical investigation was carried out in order to evaluate the electronic effects in obtaining the interaction energy of the phendione-based test compounds. The semi-empirical calculations were based on methods developed by Michael Polanyi and Henry Eyring in 1931 (Morgon and Coutinho, 2007), for coupling quantum theory to empirical data. This category of semi-empirical methods makes it possible to approach large systems containing many atoms such as proteins, DNA and other molecular systems comprising tens of thousands of atoms (Morgon and Coutinho, 2007). Therefore, the phendione-based compounds being considered in the present study, having been docked in the lasB active site, were subjected to PM6 calculations in order to obtain their interaction energy, according to Equation (4):

(4) $$E_{i\,n\,t}=E_{s\,y\,s\,t\,e\,m/i\,n\,t\,e\,r\,m}-E_{s\,y\,s\,t\,e\,m}-E_{i\,n\,t\,e\,r\,m}$$

The application of these techniques is quite important in order to acquire a better understanding of the possible interaction modes of the test compounds within the lasB active cavity.

### Microorganisms

In the present work, two *P. aeruginosa* strains were used in the experiments: a reference strain (ATCC 27853), which was formerly isolated from blood (Medeiros et al., 1971) and a bloodstream clinical isolate named 09HC (Silva et al., 2014). Both *P. aeruginosa* strains were previously characterized by our research group regarding their antimicrobial susceptibility profiles: 09HC strain was resistant to ceftazidime, meropenem and imipenem, while the ATCC strain was susceptible to the three antimicrobials (Silva et al., 2014).

### Planktonic- and Biofilm-Growing *P. aeruginosa* Cells

*P. aeruginosa* cells were grown on trypticase soy agar (TSA; Merck, Darmstadt, Germany) for 18 h at 37°C. Subsequently, planktonic bacterial cells were subcultured in tryptone soy broth (TSB) supplemented with 1% glycerol, 50 mM glutamate, 10 mM CaCl_2_ and 10 mM ZnCl_2_ and incubated for 24 h at 37°C under constant agitation (Marquart et al., 2005). Cultures were centrifuged (4000 rpm, 20 min, 4°C) and bacterial cells were washed three times in phosphate-buffered saline (PBS; 150 mM NaCl, 20 mM phosphate buffer, pH 7.2). In parallel, the spent culture supernatants were filtered through a 0.22 μm membrane (Millipore, São Paulo, Brazil). The biofilm formation assay was carried out as described previously by Silva et al. (2014). Briefly, a bacterial suspension was prepared corresponding to 0.5 McFarland turbidity scale (∼10^8^ colony-forming units/mL) in TSB medium. Next, 100 μL of the culture was pipetted into each well of a flat-bottomed 96-well polystyrene plate and incubated for 24 h at 37°C. Subsequently, biofilm supernatants were collected, combined in a single tube and filtered through a 0.22 μm membrane.

### Processing of the Supernatants for Enzymatic Measurements

The cell-free supernatants were concentrated 10-fold in a 10,000 molecular weight cut-off Centricon micropartition system (Amicon, Beverly, MA, United States). The protein concentration was determined using the method described by Lowry et al. (1951), using bovine serum albumin (BSA; Sigma-Aldrich, United States) as standard.

### LasB Activity Measurement

The activity of secreted lasB was measured using a microtiter-based assay as described by Cathcart et al. (2011), and which consisted of the proteolytic cleavage of a specific fluorogenic peptide substrate, aminobenzyl-Ala-Gly-Leu-Ala-*p*-nitrobenzylamide (Peptides International, Louisville, KY, United States), bonded to the elastase of *P. aeruginosa*. The reaction mixtures (100 μL) containing the purified lasB of *P. aeruginosa* (Elastin Products Company, Owensville, MO, United States) at 10 ng per 100 μL/well or cell-free supernatants (20 μg of protein), the peptide substrate (200 μM) and the reaction buffer (50 mM Tris–HCl, 2.5 mM CaCl_2_, pH 7.2) were incubated in the absence or in the presence of different concentrations of phendione, Ag-phendione, Cu-phendione and simple salts (AgNO_3_ and CuSO_4_.5H_2_O). The fluorescence was monitored over 20 min at 30 s intervals using a SpectraMax Gemini XPS Fluorescence Microplate Reader (Molecular Devices, LLC, United States) at an excitation wavelength of 330 ± 10 nm and an emission wavelength of 460 ± 10 nm. The inhibitory constant (K*_*i*_*) values were calculated using a derivation of the Michaelis–Menten equation employing GraphPad Prism 6.1. The Michaelis–Menten constant (K*_*m*_*) value for the substrate, required for the calculation, was experimentally determined as 13.2 μM using a Lineweaver–Burk Plot.

### Zymography Assay

The protease profile was assayed by means of sodium dodecyl sulfate-polyacrylamide gel electrophoresis (SDS–PAGE) containing 0.1% gelatin incorporated into the gel as proteinaceous substrate (Heussen and Dowdle, 1980). Gels were loaded with 40 μg of proteins per slot. After electrophoresis at a constant voltage (120 V) at 4°C, SDS was removed by incubation with 10 volumes of 2.5% Triton X-100 for 1 h at room temperature under constant agitation. In order to promote the proteolysis, the gels were incubated for 24 h at 37°C in the digestion buffer comprising 50 mM Tris–HCl, 10 mM CaCl_2_, 1 mM ZnCl_2_ and 150 mM NaCl, pH 8.0 (Marquart et al., 2005). The gels were stained for 2 h with 0.2% Coomassie brilliant blue R-250 in methanol:acetic acid:water (50:10:40) and destained overnight in a solution containing methanol:acetic acid:water (5:10:85), to intensify the digestion halos. The molecular masses of the proteases were calculated by comparison with the mobility of low molecular mass standards (Sigma-Aldrich). The gels were dried, scanned and digitally processed.

### Bacterial Treatment With Phendione-Based Compounds

In order to investigate the impact on lasB production by treating living *P. aeruginosa* cells with phendione-based compounds, bacterial cells (10^6^) were cultured in TSB medium for 24 h at 37°C with shaking in the absence and in the presence of each compound at the concentration corresponding to the ½ × MIC value, as previously established by our group (Viganor et al., 2016): phendione (3.125 μg/mL, 14.91 μM), Ag-phendione (6.25 μg/mL, 9.93 μM) and Cu-phendione (6.25 μg/mL, 6.53 μM). After the treatment period, the bacterial cultures were centrifuged in order to obtain the cell-free supernatants (which were used to detect the lasB protein by Western blotting) and the bacterial cells [which were used to analyze the expression of the *lasB* gene by real time-polymerase chain reaction (RT-PCR)].

### LasB Immunodetection

The detection of lasB extracellularly released into the culture supernatant was performed using a Western blotting assay (Marquart et al., 2005). Briefly, proteins (100 μg) present in the cell-free supernatants were mixed with SDS-PAGE sample buffer (125 mM Tris, pH 6.8, 4% SDS, 20% glycerol, 0.002% bromophenol blue and 10% β-mercaptoethanol) followed by heating at 100°C for 5 min. Proteins were then separated on 12% SDS–PAGE and the polypeptides electrophoretically transferred at 4°C at 100 V/200 mA for 2 h to a nitrocellulose membrane. Non-specific protein biding sites were blocked using 5% skimmed milk in TBS-Tween (150 mM NaCl, 10 mM Tris, 0.05% Tween 20, pH 7.4) for 1 h at room temperature under constant agitation. The membrane was washed three times in TBS-Tween and then incubated with the anti-lasB primary polyclonal antibody (kindly provided by Dr. Richard O’Callaghan, University of Mississippi Medical Center, United States), which was diluted at 1:500, for 4 h. The secondary antibody used was peroxidase-conjugated goat anti-rabbit IgG at 1:10,000, followed by chemiluminescence immunodetection using an ECL kit (GE Healthcare, Chicago, IL, United States) (Marquart et al., 2005). Glyceraldehyde 3-phosphate dehydrogenase (GAPDH) is a highly conserved protein that is constitutively secreted into the extracellular environment via vesicles released from the outer membrane of *P. aeruginosa* cells (Kim et al., 2013; Vanden Bergh et al., 2013). Based on this information, the polyclonal anti-human GAPDH antibody produced in rabbit (Sigma-Aldrich), at 1:10000 dilution, was selected and used as a loading control. Thus, in order to confirm the homology between human and pseudomonal GAPDH, we aligned the both protein sequences and then calculated the homology score using clustal2.1 (Supplementary Figure S1). The homology score is defined as the number of identities (same amino acid residue in the best alignment divided by the number of residues compared (gap positions are excluded). Thus, the comparison of the sequence of *P. aeruginosa* GAPDH reveals 47.11% homology with the human GAPDH. In addition, considering the conserved substitutions and semi−conserved substitutions in pairwise score calculations, the similarity score between human and pseudomonal GAPDH increased to 78.50%. Taken together, these data support the recognition of pseudomonal GAPDH protein by the anti-GAPDH antibody used in our experiment. The relative molecular masses of the reactive polypeptides were calculated by comparison to the mobility of low molecular mass protein standards (Sigma-Aldrich).

### RT-PCR for Detection of *LasB* Gene

Total RNA isolation was performed using a TRIzol^®^ bacterial RNA isolation kit (Thermo Fisher Scientific, Waltham, MA, United States) in accordance with the manufacturer’s instructions. The concentration of RNA was measured using Nanodrop 2000 (Thermo Fisher Scientific) and the quality was evaluated by resolving on a 1.5% agarose gel at 120 V. Real-time one-step PCR was performed in a 96-well plate on the purified RNA samples according to the QuantiFast^®^ SYBR^®^ Green RT-PCR Handbook protocol (Qiagen, Germany). Five microliters of both forward and reverse primers (1 μM) and 1 μl RNA (∼100 ng) were added to each well (final volume of 50 μL). The primers used were *lasB* (forward: 5′-AAGTGCTCGATCAGTGGGAA-3′ and reverse: 5′-CTGCTTGTAGGTGTTGGTCG-3′, designed using Primer3 Output program) and the housekeeping gene *rpoD* (forward: 5′-GGGCGAAGAAGGAAATGGTC-3′ and reverse: 5′-CAGGTGGCGTAGGTGGAGAA-3′) (Savli et al., 2003). As previously reported (Savli et al., 2003; McMillan and Pereg, 2014), the *RpoD* gene was selected because its expression is stable under stress conditions. In the present study, no significant alterations were reported in the *rpoD* gene expression in either untreated or phendione-treated cells (Supplementary Figure S2). To do this, samples were analyzed on a Light Cycler 480 (Roche Life Science, Sussex, United Kingdom) employing the following program: PCR initial activation step at 95°C for 15 min, followed by 45 cycles of 95°C for 10 s (denaturation), 63°C for 10 s (annealing) and 72°C for 10 s (extension), then cooling at 4°C. Finally, the ΔΔC_*T*_ value was used to determine the fold change in expression (Schmittgen and Livak, 2008).

### Effects of Phendione-Based Compounds on the LasB-Induced Damage to A549 Cells

A549 (human alveolar basal epithelial cell line, ATCC CCL-185) cells were maintained in 75 cm^2^ cell culture flasks containing Dulbecco’s modified Eagle medium (DMEM; Sigma-Aldrich) supplemented with 10% heat-inactivated fetal bovine serum (FBS) and 2 mM L-glutamine at 37°C in an atmosphere of 5% CO_2_. For the experiments, A549 cells were seeded (10^4^ per well) into 96- or 24-well plates containing the above medium and grown to confluence. Firstly, 50 μL of the cell-free pseudomonal supernatant (equivalent to 100 μg protein) and purified lasB (10 ng protein) were previously treated with 25 μM of each phendione-based compound for 1 h at 37°C. Then, 20 μL each of the neutralized supernatant and the neutralized purified lasB were added to the A549 monolayer in 180 μL of fresh DMEM, followed by incubation for 48 h at 37°C in an atmosphere of 5% CO_2_. In parallel, A549 cells exposed only to phendione-based compounds were used as a viability control. Cell viability was determined by the reduction of the 3-(4,5-dimethyl-2-thiazolyl)-2,5-diphenyl-2H-tetrazolium (MTT) bromide salt, and the integrity of the monolayer as well as the cell morphology were observed using light-field microscopy (Aoki et al., 2010).

### Effects of Phendione-Based Compounds on the Purified LasB- and LasB-Rich Bacterial Secretions-Induced Toxicity on *Galleria mellonella* Larvae

Larvae of *G. mellonella* were obtained at the insectarium of Biochemistry Department, Chemistry Institute from UFRJ, Brazil. Breeding and maintenance of *G. mellonella* larvae were carried out as previously described (Fernandes et al., 2017). Larvae from the last instar employed in experiments were selected after reaching similar size (15–20 mm), weight (approximately 200 mg) and by the absence of gray pigmentation (Fernandes et al., 2017). Prior to the infection assay, the supernatants of *P. aeruginosa* strains (ATCC 27853 and 09HC) were obtained, filtered and concentrated as described above. Then, groups of 10 larvae were inoculated with 10 μL of the (i) pseudomonal supernatants (containing 100 μg of protein), (ii) thermal-inactivated supernatants (100°C for 5 min) and (iii) purified LasB molecules (1 μg) by directly injection into the hemocoel via the last left pro-leg, using a 25 μL Hamilton syringe. As a physical injury control, the same volume of PBS was also inoculated into *G. mellonella* larvae. In addition, an extra control group of larvae without any inoculation were employed to evaluate the health of larvae during the follow-up of the experimental conditions. After injection, the larvae were incubated in glass dishes without food and in the dark at 37°C for up to 7 days. Survival was appraised daily at the same time as the beginning of the experiment, and the larvae were considered as being dead when no reaction was observed upon stimulus (Fernandes et al., 2017; Rizzo et al., 2017). Survival was expressed as the percentage of living larvae along the experimental time (Fernandes et al., 2017; Rizzo et al., 2017). In parallel, we investigated the ability of Cu-phendione to prevent larvae death induced by purified LasB and by LasB-rich bacterial secretions. To do this, different concentrations (1, 10, and 100 μg of protein) of the bacterial supernatant obtained from ATCC 27853 strain and the purified LasB (1 μg) were previously treated with 50 μM Cu-phendione solution for 30 min at room temperature and, subsequently, 10 μL were inoculated into the larvae and survival rates were monitored daily for 7 days.

### Statistics

Experiments were performed in triplicate, in three independent experimental sets, and data were expressed as mean ± standard deviation. The results were evaluated by analysis of variance (ANOVA) and Dunnett’s multiple comparison test using GraphPad Prism 6 computer software. In *in vivo* experiments, statistical significance was plotted using Kaplan–Meier curves on GraphPad Prism 7.0 software. In all analyses, *P*-values of 0.05 or less were considered statistically significant.

## Results

### Docking of the Phendione-Based Compounds With the LasB-Encapsulated HPI/LasB Active Site: *In silico* Approaches

**FIGURE 2:**
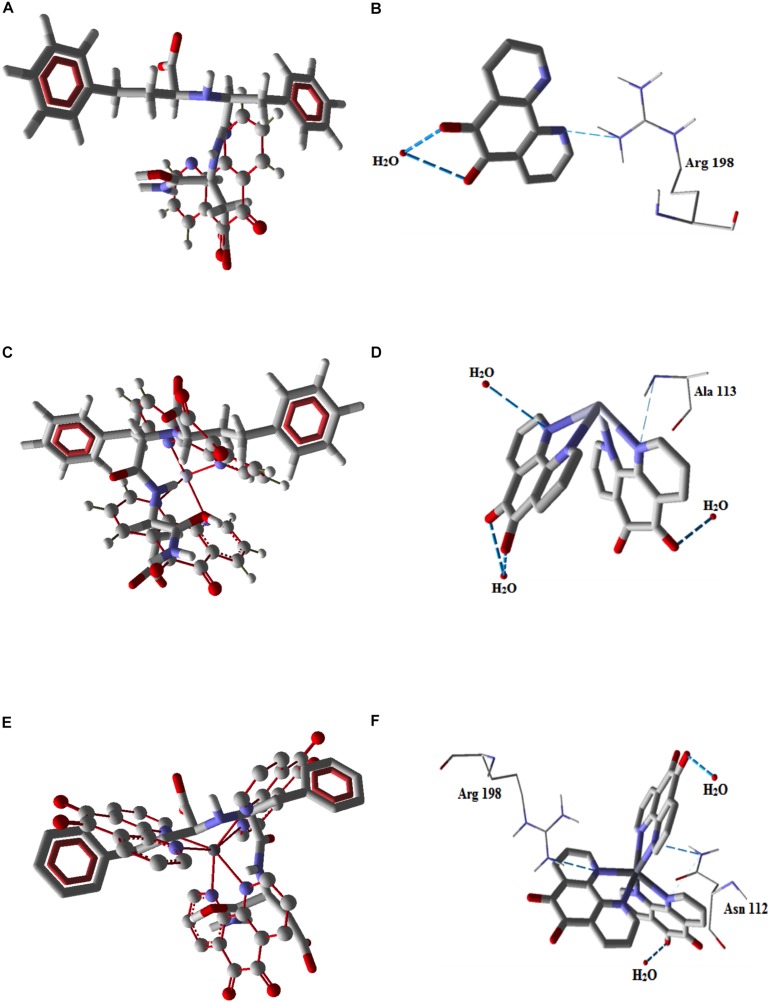
Molecular docking diagrams showing the bonding interactions between the phendione compounds and the lasB-encapsulated HPI/lasB active site of *P. aeruginosa.* Docking of phendione **(A)**, Ag-phendione **(C)** and Cu-phendione **(E)** with the *N*-(1-carboxy-3-phenylpropyl)phenylalanyl-α-asparagine moiety illustrated using ball and stick models, and π-stacking interactions are highlighted in red dashed lines. Hydrogen bonds between lasB, water molecules and phendione **(B)**, Ag-phendione **(D)** and Cu-phendione **(F)** are shown with blue dashed lines.

The possible interactions between the phendione-based compounds and the lasB-encapsulated HPI/lasB active site were investigated using molecular docking methods (Figure 2 and Table 1). The docking of the phendione-based test compounds to the lasB active inhibitor ligand, HPI, revealed that Cu-phendione adhered firmly to HPI via π-stacking interactions between aromatic rings (Figures 2E,F). Furthermore, the docking calculations showed that Cu-phendione had the greatest interaction energy (−127.54 kcal mol^–1^), followed by phendione (−40.47 kcal mol^–1^) and Ag-phendione (−34.26 kcal mol^–1^) (Table 1). Corroborating these findings, it was also observed that Cu-phendione was able to form two hydrogen bonds with amino acid residues (Arg_198_ and Asn_112_) in the lasB catalytic site, as well as two other hydrogen bonds with water molecules (Figures 2E,F and Table 1). Likewise, Ag-phendione established interactions with the lasB catalytic site by forming one hydrogen bond with one amino acid residue (Ala_113_) and three water molecules (Figures 2C,D and Table 1), while phendione interacted only moderately with the lasB active site forming just two hydrogen bonds (with Arg_198_ and one water molecule; Figures 2A,B and Table 1).

**TABLE 1:** Results of the *in silico* analyses showing the interaction energies of the phendione-based compounds with the lasB-encapsulated HPI/lasB active site.

	**Molegro Virtual Docker**		**Semi-empiric – PM6**
**Compounds**	**Interaction energy (kcal mol^–1^)**	**Hydrogen bonds**	**Interaction energy (kcal mol^–1^)**
Phendione	−40.47	1 Arg_198_ 1 H_2_O	4.179
Ag-phendione	−34.26	1 Ala_113_ 3 H_2_O	2.690
Cu-phendione	−127.54	1 Arg_198_ 1 Asn_112_ 2 H_2_O	0.000

In addition, semi-empirical calculations were performed at PM6 level in order to obtain more precise values of their hydrogen bonding interaction energies with amino acid residues within the cavity of the lasB enzyme, and comparing these new findings with those obtained by the docking analyses. In agreement with the docking data, Cu-phendione presented the greatest relative interaction energy (0 kcal mol^–1^), followed by Ag-phendione (2.690 kcal mol^–1^) and phendione (4.179 kcal mol^–1^) (Table 1). The interaction energies of phendione and Ag-phendione were quite similar (energy difference 1.489 kcal mol^–1^) and a parallel trend was also observed from the docking studies. Based on these findings, it is evident that phendione and its metal-based complexes could actively interact with lasB. Given that Cu-phendione presented the best fit to the HPI/lasB active site model and also had the greatest interaction energy, this metal complex presents the most favorable attributes for interaction with the *P. aeruginosa* lasB catalytic site.

### Effects of Phendione-Based Compounds on the LasB Enzymatic Activity

**FIGURE 3:**
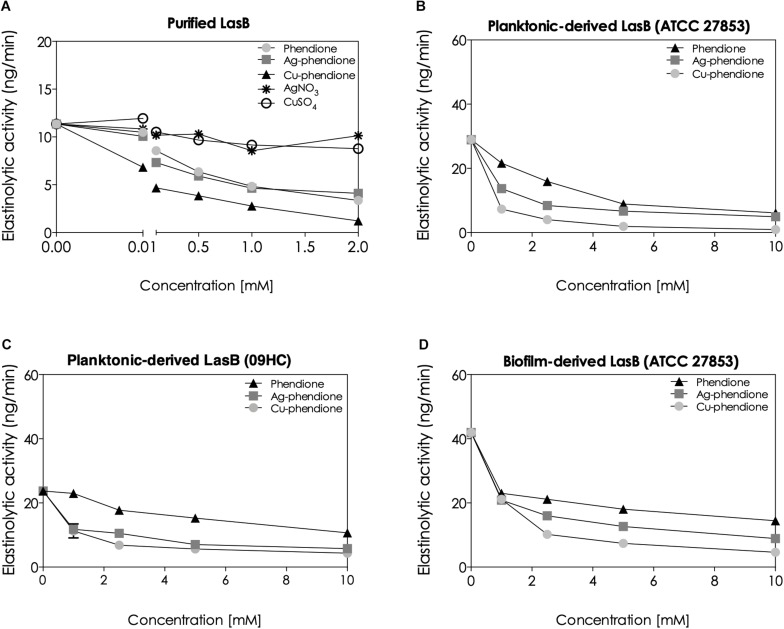
Effects of the phendione-based compounds on the elastinolytic activity of *P. aeruginosa*. The enzymatic activity was measured using a specific fluorogenic peptide substrate to *P. aeruginosa* elastase, taking into consideration the following experimental systems: purified pseudomonal lasB molecules **(A)**, supernatants obtained from planktonic growth of two *P. aeruginosa* strains, ATCC 27853 **(B)** and 09HC **(C)**, and supernatant-derived from the biofilm of the ATCC 27853 strain **(D)**. Before the enzymatic quantification, each system was treated for 30 min with different concentrations (0, 0.001, 0.01, 0.1, 0.5, 1, and 2 mM for the purified lasB, and 1, 2.5, 5, and 10 mM for the conditioned bacterial supernatants) of the phendione-based compounds or simple Ag^+^ and Cu^2+^ salts.

Following the *in silico* analyses, the *in vitro* impact of the phendione compounds on the enzymatic activity of lasB was investigated using a specific fluorogenic peptide substrate (Cathcart et al., 2011). In support of the above *in silico* results, Cu-phendione had the greatest inhibitory action on the purified lasB [having the lowest inhibitory constant value (K*_*i*_* = 0.09 μM)], followed by Ag-phendione (K*_*i*_* = 0.31 μM) and phendione (K*_*i*_* = 0.38 μM) (Figure 3). The simple salts, CuSO_4_.5H_2_O and AgNO_3_, did not significantly alter the elastinolytic activity (Figure 3).

It is well known that lasB is physiologically released into the extracellular environment by *P. aeruginosa* cells (Galdino et al., 2017; Azam and Khan, 2018). As expected, phendione, Ag-phendione and Cu-phendione were able to block the proteolytic activity of lasB present in the supernatants harvested from planktonic-growing *P. aeruginosa* cells (ATCC 27853 and 09HC) (Figure 3). Cu-phendione at a concentration of 10 mM, inhibited, respectively, 95.18 and 93.44% of the lasB activity detected in the secretions of planktonic-growing bacterial cells of both ATCC 27853 and 09HC strains (Figures 3B,C). Furthermore, Cu-phendione robustly inhibited (∼90%) of the lasB activity in supernatants recovered from ATCC 27853 biofilm-growing cells (Figure 3D).

### Effect of Phendione-Based Compounds on *LasB* Gene Expression and LasB Protein Secretion

**FIGURE 4:**
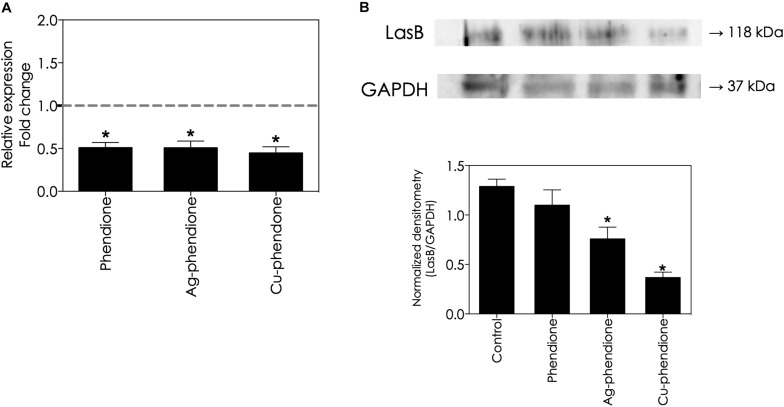
Effects of phendione-based compounds on *P. aeruginosa lasB* gene expression and lasB protein production. Bacterial cells were treated with 0.5 × MIC of phendione (16.15 μM), Ag-phendione (7.02 μM), and Cu-phendione (3.88 μM) for 24 h at 37°C. Evaluation of *lasB* gene expression by RT-PCR (*rpoD* gene was used as a housekeeping gene) **(A)**. Detection of production/secretion of lasB protein by western blot using an anti-lasB antibody (GAPDH was used as loading control) **(B)**. In **(B)**, the numbers on the right indicate the molecular masses of the reactive proteins. Reactive bands were densitometrically quantified using the ImageJ software and the results were expressed as a correlation between lasB and GAPDH densitometric values (expressed as arbitrary units). The symbols (*P < 0.05, one-way ANOVA, Dunnett’s multiple comparison test) denote the statistically difference among phendione-treated systems and untreated ones.

In this set of experiments, we evaluated whether phendione-based compounds would be able to control *lasB* gene expression, as well as the production of its mature protein product, the lasB secreted form. To do this task, live *P. aeruginosa* cells were subjected to sub-inhibitory concentrations (values corresponding to ½ × MIC) of the test compounds for a 24 h period. Phendione and its metal-containing complexes down-regulated the *lasB* gene expression in a similar way, reducing it by approximately 50% compared with the untreated cells (Figure 4A). In addition, the amount of lasB protein present in the planktonic supernatant was significantly reduced following treatment with sub-inhibitory concentrations of phendione (14.70%), Ag-phendione (41.14%) and Cu-phendione (71.43%), compared with the untreated bacterial cells (Figure 4B).

### Effect of Phendione-Based Compounds on LasB-Induced Cell Damage

**FIGURE 5:**
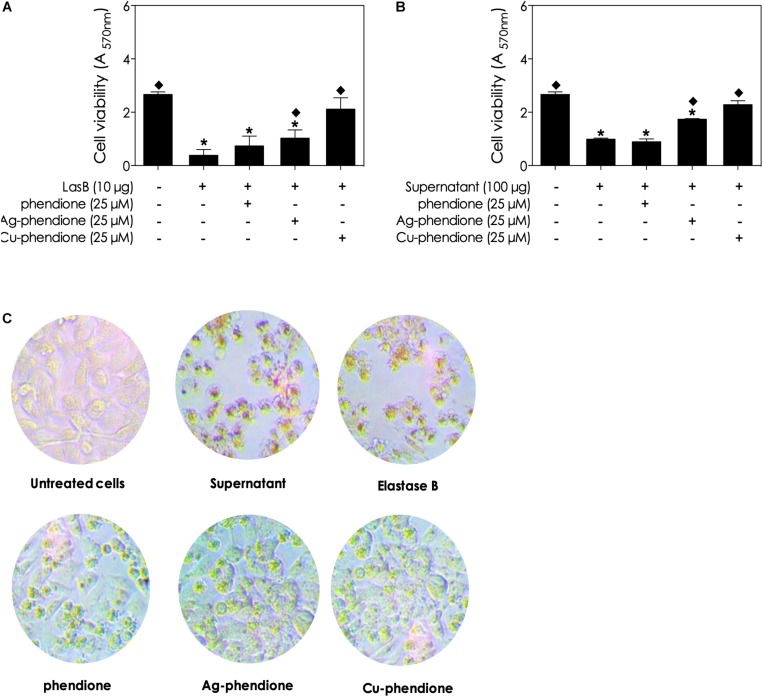
Effects of phendione-based compounds on *P. aeruginosa* lasB-induced cellular damage to lung epithelial cells (A549 line). Purified lasB **(A)** and pseudomonal conditioned supernatant **(B)** were previously incubated with phendione-based compounds at 25 μM for 1 h. Then, the lung epithelial cells were incubated for 48 h in the presence of *P. aeruginosa* supernatant (100 μg protein) or purified lasB (10 ng protein). After 48 h of incubation, A549 viability was analyzed using the MTT assay **(A,B)**. In parallel, the integrity of the A549 monolayer and cell morphology were observed by light microscopy **(C)**. The asterisks (*) denote significant difference between non-treated and treated cells (*P* < 0.05, ANOVA – Dunnett’s multiple comparison test), setting the untreated cells as a comparative parameter. 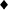 < 0.05 – One-way ANOVA (Dunnett’s multiple comparison test), setting the supernatant-treated cells as a comparative parameter.

During tissue invasion by *P. aeruginosa* cells, the bacterial lasB enzyme dislocates mammalian cellular layers by degrading extracellular components (e.g., collagen types III and IV, laminin, fibronectin and vitronectin) (Beaufort et al., 2013; Reboud et al., 2016) and disrupts several tight junctions (e.g., occludin, claudin-1 and -4, and tricellulin) (Beaufort et al., 2013; Nomura et al., 2014). Thus, it would be expected that the inhibition of bacterial lasB activity by phendione-based compounds would result in the attenuation of lasB-related mammalian cell damage. With this proposition in mind, it was observed that the incubation of a A549 monolayer with either culture supernatants (100 μg of protein) or purified lasB (10 μg) for 48 h reduced cell viability (Figures 5A,B) and induced cell rounding and detachment (Figure 5C). In addition, in the presence of either conditioned culture supernatant or purified lasB from *P. aeruginosa*, the A549 viability decreased 37.03 and 14.38%, respectively, in comparison with non-treated epithelial cells (Figures 5A,B). In contrast, addition of the phendione-based compounds (at 25 μM) protected epithelial cells from lasB-related cytotoxic effects, based on the maintenance of A549 morphology and monolayer confluence (Figure 5C). Furthermore, A549 viability was partially restored in the presence of planktonic bacterial supernatant previously incubated with phendione, Ag-phendione and Cu-phendione (Figure 5B). Likewise, the treatment of purified lasB molecules with phendione, Ag-phendione and Cu-phendione reduced its ability to affect the growth of A549 cells by approximately 28, 39, and 80%, respectively, compared to A549 cells treated with lasB (Figure 5A). Finally, under the experimental conditions employed herein, phendione, Ag-phendione and Cu-phendione alone were not toxic to A549 cells when administered up to a concentration of 25 μM (data not shown).

### Effects of Phendione-Based Compounds on the Purified LasB- and LasB-Rich Bacterial Secretions-Induced Toxicity on *G. mellonella* Larvae

**FIGURE 6:**
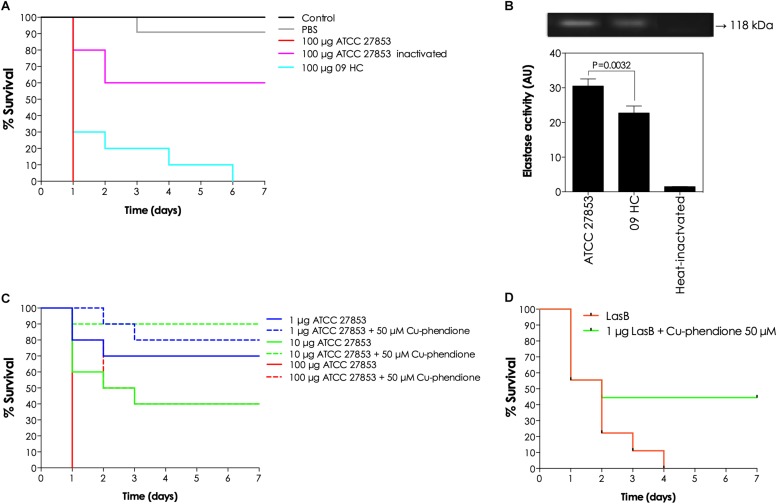
*In vivo* effects of phendione-based compounds on the lasB-induced toxicity against *G. mellonella* larvae. Firstly, the toxic effects of cell-free culture supernatants obtained from ATCC 27853 and 09HC were evaluated. In this way, fresh and thermally inactivated (100°C/5 min) bacterial supernatants (100 μg) were injected into the larvae hemocoel (10 per group), and the larvae survivability was followed up for 7 consecutively days **(A)**. The presence of lasB in the pseudomonal supernatants was confirmed by the cleavage of the specific fluorogenic peptide substrate (aminobenzyl-Ala-Gly-Leu-Ala-*p*-nitrobenzylamide) and by gelatin-zymography. The molecular mass on the right evidences the elastase proteolytic band. Note the significant difference regarding the elastase production by both *P. aeruginosa* strains **(B)**. Subsequently, Cu-phendione was tested on its ability to revert the toxic effects of both bacterial supernatants **(C)** and purified lasB molecules **(D)**. In this set of experiments, different concentrations (1, 10, and 100 μg of proteins) of *P. aeruginosa* ATCC 27853 secretions and purified lasB molecules (1 μg) were treated or not with 50 μM Cu-phendione for 30 min at room temperature. Then, both untreated and Cu-phendione-neutralized systems were injected into larvae hemocoel (10 per group), and the larvae survival was checked daily for 7 days. The survival data were plotted using the Kaplan–Meier method using GraphPad Prism v 7.0.

Given the inhibitory activity of phendione-based compounds on *in vitro* lasB enzymatic activity, the efficacy of Cu-phendione (the most potent lasB inhibitor) on the attenuation of the toxicity of pseudomonal supernatant was explored in an *in vivo* infection model using the larvae of the insect, *G. mellonella.* Firstly, the protective ability of Cu-phendione was tested against the *in vivo* toxicity mediated by the supernatants obtained from the ATCC 27853 and 09HC strains (Figure 6A). The pseudomonal secretions from the resistant strain (09HC) exhibited an attenuated virulence profile on *G. mellonella* larvae compared with the reference strain ATCC 27853. In a comparative perspective, 100 and 70% of the larvae inoculated with ATCC- and 09HC-obtained supernatants, respectively, died after the first 24 h of inoculation (Figure 6A). Further, the heated-supernatant from the ATCC strain (a classical enzymatic inactivation process) was considerably less toxic to the larvae compared with the fresh, non-heated counterpart (Figure 6A). Interestingly, lasB production was significantly different for the two bacterial strains (as judged either by the cleavage of a *P. aeruginosa* elastase-specific peptide substrate or by gelatin-containing zymography assays), with the ATCC strain producing around 1.4-times more lasB than the 09HC strain (Figure 6B).

Since the ATCC 27853-derived supernatant was more toxic toward *G. mellonella*, this sample was selected in order to assess the possible beneficial effects of Cu-phendione in the *in vivo* scenario. Different supernatant concentrations were previously incubated in the absence or presence of Cu-phendione (50 μM) for 30 min and then injected into the larvae. It was found that the *P. aeruginosa* supernatant was noxious to *G. mellonella* larvae in a typical dose-dependent manner (Figure 6C). Interestingly, Cu-phendione mitigated the toxic effect of lasB-containing bacterial secretions, increasing the survival rate of the *G. mellonella* larvae (Figure 6C). In addition, purified lasB protein was also able to induce larval death and Cu-phendione exerted a protective effect against this toxic action (Figure 6D). Cu-phendione alone was not toxic to *G. mellonella* larvae in concentrations up to 50 μM (data not shown).

## Discussion

Considering the worldwide crisis of bacterial resistance, *P. aeruginosa* is a constant threat in relation to community- and hospital-acquired infections, primarily because of the emergence and quick spread of refractory strains that are unresponsive to every clinically available antimicrobial drug (Strateva and Yordanov, 2009). So, the arising and foreseeable failure of classical antimicrobial therapies have spurred the scientific community to develop new strategies to tackle MDR pathogens (Dickey et al., 2017).

*Pseudomonas aeruginosa* possesses a devastating suite of virulence attributes, which act together to enhance the ability of the pathogen to cause host cell damage and, consequently, to aggravate the infectious process and disease course (Balasubramanian et al., 2013). For this reason, the purpose of targeting the bacterial virulence arsenal is a veritable and promising approach to the development of alternative drugs, which act by attenuating the aggressiveness of the pathogen and by reducing its ability to cause a vigorous infection (Dickey et al., 2017). Since this anti-virulence approach does not target main, vital bacterial pathways, these new therapeutics impose less selective pressure than the classical antimicrobial drugs, so preventing or delaying the emergence of the drug-resistant phenotype (Dickey et al., 2017). Moreover, the majority of virulence factors are specific to either a single or a few closely similar species. Thereby, the horizontal gene transfer of resistance genetic elements of an anti-virulence drug would be reduced (Totsika, 2016).

In recent times, our group has been exploring the antimicrobial potential of 1,10-phenanthroline- and phendione-based compounds (McCann et al., 2012a; Viganor et al., 2017). In this sense, we previously reported that the coordination of the phendione ligand to transition metals represents a new and promising group of antibiotic drugs, in which both Ag-phendione and Cu-phendione have exhibited a striking anti-*P. aeruginosa* effect against both planktonic- and biofilm-forming cells (Viganor et al., 2016). Moreover, these compounds were able to kill metronidazole-resistant *Trichomonas vaginalis* clinical isolates (Rigo et al., 2018) and they were active against several fungi such as *Phialophora verrucosa*, *Scedosporium apiospermum*, *Candida albicans*, and *Candida haemulonii* (McCann et al., 2012a; Gandra et al., 2017; Granato et al., 2017). Herein, we investigated the applicability of phendione-based compounds as anti-virulence drugs through the blockage of pseudomonal lasB activity.

LasB belongs to the metalloprotease class of enzymes (Zn^2+^ and Ca^2+^ cofactors), and thus the addition of metal-chelator molecules (ligands) with the capability of abstracting the central metal ion from the protein are expected to inhibit its enzymatic activity. Accordingly, multidentate EDTA and EGTA prevent lasB enzymatic activity by sequestering both Zn^2+^ and Ca^2+^ ions that are essential for its proteolytic activity and its three-dimensional structure, respectively (Kocabiyik et al., 1995; Aoki et al., 2010). In contrast, phendione-based compounds may inhibit the elastinolytic activity of lasB by blocking the access of the host substrate to the active site. LasB has the ability to cleave a broad variety of proteinaceous substrates, hydrolyzing internal peptide bonds of proteins specifically on the amino portion of hydrophobic amino acid residues (Miyoshi and Shinoda, 2000). Furthermore, the S_1_’ sub-site within the active site is a deep hydrophobic pocket that accepts bulky aromatic and large aliphatic chain groups (Carson et al., 2012). For this reason, it is possible that the hydrophobic skeleton of the phendione-based compounds could interact with that section of the lasB active site.

Using *in silico* approaches, it was observed that phendione and its metal complexes overlapped with the lasB active and standard ligand, HPI. Cu-phendione, in particular, encompassed compatible structural moieties of the HPI, principally π-stacking interactions between the aromatic rings. HPI binds to the active site in the S_1_-S_1_’ sub-sites of lasB by hydrogen bonding, hydrophobic and weak van der Waal’s interactions (Lutfullah et al., 2008). Given the apparent compatibility of the phendione-based compounds with HPI, it is conceivable that they may interact with the lasB active site in a similar fashion, with the trend: Cu-phendione > Ag-phendione > phendione. Zhu et al. (2015) described mercaptoacetamide-derivative compounds as an effective class of lasB inhibitors, and the docking analysis showed that the amide portion of mercaptoacetamide compounds interact with Asn_112_ residue and the phenyl ring shielding some of the hydrophobic residues consisting of Phe_129_, Leu_132_, Val_137_, Ile_190_, and Leu_197_, blocking the enzymatic activity of lasB. Likewise, the current *in silico* study suggested that Cu-phendione was able to interact with the *P. aeruginosa* lasB sub-site S_1_ by binding to Arg_198_ and Asn_112_.

After evaluating the potential molecular interactions between pseudomonal lasB and the phendione compounds, the enzyme inhibition effectiveness was confirmed by *in vitro* measurements. It was observed that phendione and its Ag^+^ and Cu^2+^ coordination complexes were able to completely inhibit the proteolytic activity of purified lasB from *P. aeruginosa* in a typical dose-dependent fashion. Moreover, the test compounds inhibited the lasB elastinolytic activity in the supernatants obtained from bacterial cells grown under both planktonic and biofilm lifestyles. As predicted by the molecular docking studies, Cu-phendione was the compound best able to block the *P. aeruginosa* lasB enzymatic activity.

Given the potential of lasB as a target for the development of a new anti-virulence drug, several other research groups have described the inhibitory action of natural and/or synthetic molecules on the lasB activity of *P. aeruginosa*. It was reported that sub-inhibitory concentrations of some FDA-approved antibiotics modulated the *P. aeruginosa* virulence attributes. For instance, erythromycin, at a concentration of 8 μg/mL, abolished the elastinolytic activity of 20 out of 34 (58.5%) *P. aeruginosa* clinical strains recovered from the sputum of patients suffering from various airway infections (Sakata et al., 1993). It was also observed that treatment with a sub-inhibitory concentration of doxycycline (4 μg/mL) decreased the secretion of lasB by 67% (Husain and Ahmad, 2013). Moreover, the administration of a sub-inhibitory concentration of tobramycin (0.063 mg/L) combined with bismuth-ethanedithiol (0.1 μM) decreased lasB production by 70% in the *P. aeruginosa* PAO1 reference strain (Alipour et al., 2010). It was reported that analogs of 3-hydroxy-1-alkyl-2-methylpyridine-4(1H)-thiones effectively blocked the lasB activity of the *P. aeruginosa* PA14 strain, exhibiting IC_50_ values of approximately 3 μM (Garner et al., 2012). Also, the synthetic *N*-α-mercaptoacetyl dipeptides, HS-CH_2_-CO-Phe-Tyr-NH_2_ and HS-CH_2_-CO-Trp-Tyr-NH_2_, showed high activity against lasB, presenting K*_*i*_* values of 41 and 40.5 nM, respectively (Cathcart et al., 2011). The valuable application of metal nanoparticles to anti-virulence therapy has also been highlighted, again due to their anti-lasB attributes. Nanoparticulate zinc oxide (at 200 μg/mL) completely blocked pseudomonal elastinolytic activity, besides offering protection against elastase-induced tissue destruction and epithelial ulceration in rabbit skin burns (Ali et al., 2017). Likewise, an association of selenium nanoparticles with polyphenols obtained from honey (at 4.5 μg/mL) inhibited 52.7% of the pseudomonal lasB activity of the PAO1 reference strain (Prateeksha et al., 2017). Furthermore, metabolites derived from the fungus *Rhizopus arrhizus*, when associated with silver nanoparticles (at 25 μg/mL), repressed *lasB* gene expression in *P. aeruginosa* by 84% (Singh et al., 2015). Our current investigation revealed that Ag-phendione and Cu-phendione were powerful lasB inhibitors, presenting inhibition constant (K*_*i*_*) values of 0.31 and 0.09 μM, respectively. Additionally, treating *P. aeruginosa* cells with sub-inhibitory concentrations of the phendione-based compounds repressed the expression of the *lasB* gene as well as preventing the production and/or secretion of mature lasB protein. The molecular mechanisms underlying reduced *lasB* gene expression and other virulence factors in *P. aeruginosa* is due to the QS regulatory networks. It is well known that QS regulatory networks respond to environmental stress cues (including inhibitors and other chemical compounds), and the two main regulatory networks are *las* and *rhl* gene systems that activate the expression of QS-responsive genes (and proposed to constitute nearly 10% of the *P. aeruginosa* genome). However, more recent studies have reported the existence of two additional QS networks (quinolone-based and integrated QS) that are integrated and interconnected with the *las* and *rhl* systems (Lee and Zhang, 2015). Therefore, we suggest that the decrease in *lasB* expression observed in the current investigation is most likely as a consequence of the phendione-based compounds exerting their effects on the QS networks. This line of investigation is complex and time-consuming, but it is in progress by our research group.

The invasiveness of *P. aeruginosa* throughout host tissues is mediated by the action of lasB (Reboud et al., 2016). LasB promotes the degradation of extracellular matrix proteins, aside from cleaving some essential cell-to-cell and cell-to-matrix adhesion receptors important for the maintenance of the integrity of the endothelial barrier (Beaufort et al., 2013). Consequently, at least in theory, the inhibition of lasB activity could negate its cytotoxic effects and therefore attenuate the pathogenesis of a *P. aeruginosa* infection. In this context, the protective effectiveness of several anti-lasB molecules has been reported. The co-incubation of *P. aeruginosa* culture supernatant with Ca-EDTA curtailed the destructive effect of lasB on the A549 line, restoring 26.5% of mammalian cell viability upon addition of a solution of Ca-EDTA (8 μg/mL) (Aoki et al., 2010). It was also demonstrated that phosphoramidon, a classical lasB inhibitor, delayed elastase-related cornea damage for a period of 12 h (Kawaharajo et al., 1982). Diethylene triamine penta-acetic acid (DTPA) has also been proposed as a lasB inhibitor, since a 20 μM solution significantly decreased lasB production in the *P. aeruginosa* PAO1 strain (Gi et al., 2014). Moreover, the treatment of PAO1 cells with 50 μM DTPA reduced the amount of lasB in the pseudomonal supernatant. Thus, DTPA-treated cells produced a supernatant that was non-toxic toward lung cells (Gi et al., 2014). The present experiments have demonstrated that phendione and its metal-based compounds, at a concentration of 25 μM, also have the ability to block the cytotoxic effect of lasB on lung epithelial cells.

The usefulness of invertebrate model hosts has become popular amongst the research community due to their numerous advantages over mammalian models, which includes ethical, logistical and budgetary features (Pereira et al., 2018). Moreover, since invertebrates are multicellular organisms with differentiated tissues and distinct organs, this sort of *in vivo* model could provide more robust results that may help more in the understanding of the biological effects of drug treatment (Pereira et al., 2018). In this scenario, *G. mellonella* is a convenient tool for investigating microbial pathogenesis and host responses, as well as helping in the understanding of the antimicrobial activity of novel drug candidates (Kavanagh and Sheehan, 2018). In the present study, the lasB-related deleterious effect on *G. mellonella* larvae was measured, as well as the ability of Cu-phendione to neutralize the toxic effect induced by pseudomonal secretions that are rich in LasB. The results suggested that ATCC 27853 and 09HC strains employed different virulence strategies when infecting *G. mellonella* as well as producing different degrees of LasB activity. Along the same line, Andrejko et al. (2013, 2014) compared the virulence profiles of the reference strain ATCC 27853 and two clinical isolates of *P. aeruginosa* on *G. mellonella* (Andrejko et al., 2013, 2014). Those authors reported that even though all of the *P. aeruginosa* strains were able to produce and secrete proteases, the elastase production were more relevant to the ATCC 27853-infected larvae (Andrejko et al., 2013, 2014). Inoculation of ATCC 27853 supernatant promptly activated the phenoloxidase system in the *G. mellonella* larvae, which plays a crucial role in the immunological response of the insect (Andrejko et al., 2013). Moreover, the ATCC 27853 supernatant promoted a stronger degradation of hemolymph polypeptides in comparison to the other clinical isolates (Andrejko et al., 2014). In another interesting study by Hwang and Yoon (2019), it was demonstrated that antibiotic-resistant *P. aeruginosa* strains were significantly less virulent in BALB/c mice than antibiotic-susceptible strains (considering both the reference PAO1 strain and recent clinical isolates). Those authors correlated a lower virulence profile of antibiotic-resistant *P. aeruginosa* strains with their notably poor lasB production. Since the ATCC 27853 supernatant was more toxigenic to *G. mellonella* larvae, we selected this strain to further investigate the *in vivo* anti-virulence effects of Cu-phendione. It was observed that larvae inoculated with pseudomonal supernatant exhibited lower survival rates when compared to the larvae that were injected with a Cu-phendione-neutralized supernatant. Aoki et al. (2010) showed that pseudomonal supernatant injected into the lungs of mice induced death after 5 days. Contrarily, mice infected with a Ca-EDTA-neutralized supernatant showed a significant increase in survival rate in comparison to mammals which were given the untreated-supernatant (Aoki et al., 2010). Furthermore, considering the devastating role of lasB in *P. aeruginosa* pathogenesis, the inhibition of this enzyme could possibly suppress the successful establishment of the bacterial infection. Previous studies have revealed that the deletion of *lasB* gene culminates in a less invasive infection in rabbit corneal epithelial cells (Cowell et al., 2003). Likewise, *in vitro* assays demonstrated that mice and *C. elegans* infected by the Δ*lasB* strain of *P. aeruginosa* had less severe infections when compared to the wide-type strain (Tan et al., 1999).

## Conclusion

In conclusion, the strategic synthesis of new drug molecules that target the inhibition of lasB is a desirable goal. In this light, we have now identified Ag-phendione and Cu-phendione as powerful lasB inhibitors. Both of these metal complexes were able to (i) interact with the active site of lasB, (ii) decrease the activity of purified lasB and secreted lasB obtained from bacterial supernatants, (iii) inhibit the *lasB* gene expression and (iv) block the production of secreted lasB. Additionally, due to their ability to inhibit lasB, these compounds nullified the lasB cytotoxic effects on lung epithelial cells. Interestingly, earlier *in vivo* experiments demonstrated that Ag-phendione and Cu-phendione are well tolerated by *G. mellonella* larvae and Swiss mice (McCann et al., 2012b). Importantly, we also found that phendione-based compounds did not induce chronic toxicity, maintaining the activity of aspartate aminotransferase and alanine aminotransferase in treated mice at the same level as that found in the untreated animal (McCann et al., 2012b). Corroborating these previously findings, the current study has shown that a Cu-phendione-neutralized supernatant promotes a significant increase in the survival rate of *G. mellonella* larvae challenged with *P. aeruginosa* secretions. Considering the *in vitro* and *in vivo* LasB-inhibitory activity of Cu-phendione, coupled with its low toxicity profile in both *in vitro* and *in vivo* models, strongly suggests that this Cu^2+^ compound is a promising anti-virulence therapeutic for the treatment of antibiotic-resistant *P. aeruginosa* infections.

## Data Availability

The raw data supporting the conclusions of this manuscript will be made available by the authors, without undue reservation, to any qualified researcher.

## Author Contributions

All authors conceived and designed the experiments. AG, LV, AC, EC, TM, LM, MO, and TR performed the experiments. All authors analyzed the data. MP, MH, OH, MD, MM, TR, MB, and AS contributed to reagents, materials, and analysis tools. All authors wrote and revised the manuscript, contributed to the research and approved the final version of the manuscript, and agreed to be accountable for all aspects of the work.

## Conflict of Interest Statement

The authors declare that the research was conducted in the absence of any commercial or financial relationships that could be construed as a potential conflict of interest.

## Supplementary Material

FIGURE S1 Schematic representation of protein sequences of both human and pseudomonal glyceraldehyde 3-phosphate dehydrogenase (GAPDH). In order to confirm the homology between human and pseudomonal GAPDH, we aligned the both protein sequences and then calculated the homology score using clustal2.1. The homology score is defined as the number of identities (same amino acid residue in the best alignment divided by the number of residues compared (gap positions are excluded). Thus, the comparison of the sequence of *P. aeruginosa* GAPDH indicates only moderate homology with human GAPDH (47.11%). However, considering conserved substitutions and semi-conserved substitutions in pairwise score calculations, the similarity score between human and pseudomonal GAPDH increased to 78.50%. Thus, the anti-human GAPDH antibody was able to proper detect the pseudomonal GAPDH.

FIGURE S2 Evaluation of *P. aeruginosa rpoD* gene expression by RT-PCR. The expression level of *rpoD* gene was used as normalization factor of raw values of *lasB* gene expression. For that, bacterial cells were treated with 0.5 × MIC of phendione (16.15 μM), Ag-phendione (7.02 μM) and Cu-phendione (3.88 μM) for 24 h at 37°C. Subsequently, the RNA was extracted and the levels of *rpoD* was measured. It was observed that the treatment with phendione-derivate compounds did not altered the *rpoD* gene expression.
