# Supplementary figures and images for: Disarming Pseudomonas aeruginosa Virulence by the Inhibitory Action of 1,10-Phenanthroline-5,6-Dione-Based Compounds: Elastase B (LasB) as a Chemotherapeutic Target
Source: Front Microbiol. 2019 Aug 2;10:1701. doi: 10.3389/fmicb.2019.01701 (PMC6688126; doi:10.3389/fmicb.2019.01701)

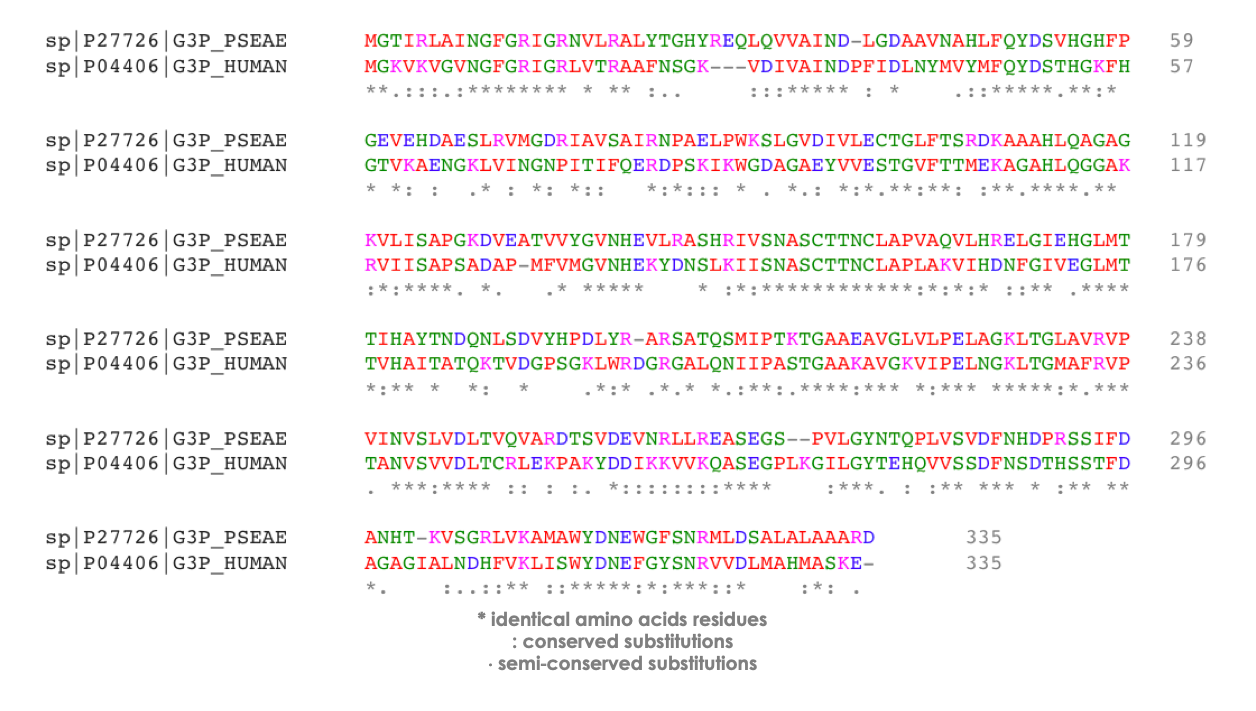

Supplement: FIGURE S1 — Schematic representation of protein sequences of both human and pseudomonal glyceraldehyde 3-phosphate dehydrogenase (GAPDH). In order to confirm the homology between human and pseudomonal GAPDH, we aligned the both protein sequences and then calculated the homology score using clustal2.1. The homology score is defined as the number of identities (same amino acid residue in the best alignment divided by the number of residues compared (gap positions are excluded). Thus, the comparison of the sequence of P. aeruginosa GAPDH indicates only moderate homology with human GAPDH (47.11%). However, considering conserved substitutions and semi-conserved substitutions in pairwise score calculations, the similarity score between human and pseudomonal GAPDH increased to 78.50%. Thus, the anti-human GAPDH antibody was able to proper detect the pseudomonal GAPDH. [file Image_1.TIFF]

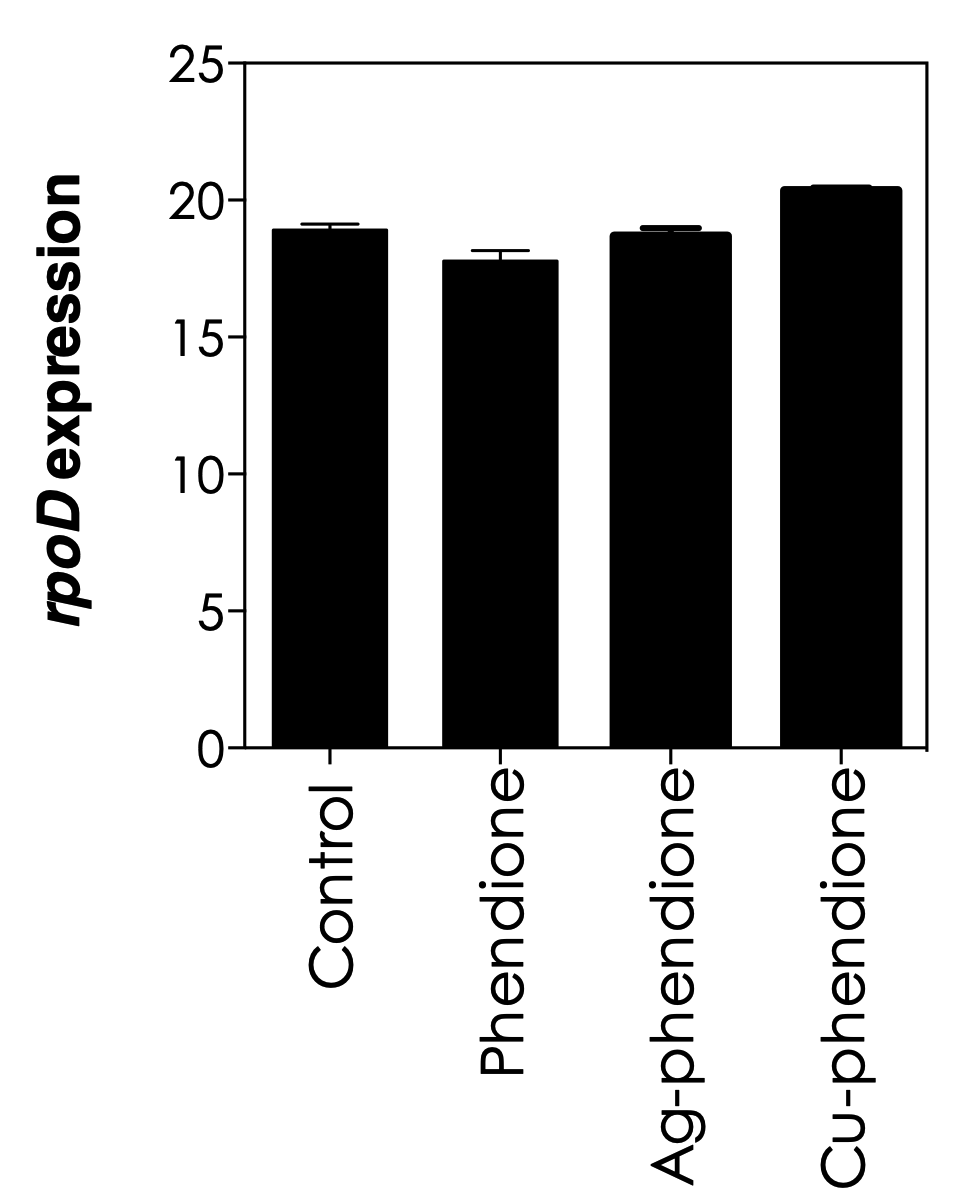

Supplement: FIGURE S2 — Evaluation of P. aeruginosa rpoD gene expression by RT-PCR. The expression level of rpoD gene was used as normalization factor of raw values of lasB gene expression. For that, bacterial cells were treated with 0.5 × MIC of phendione (16.15 μM), Ag-phendione (7.02 μM) and Cu-phendione (3.88 μM) for 24 h at 37°C. Subsequently, the RNA was extracted and the levels of rpoD was measured. It was observed that the treatment with phendione-derivate compounds did not altered the rpoD gene expression. [file Image_2.TIFF]
